# Impact of *Panax notoginseng* Residue on Rumen Microbial Community, Blood Biochemical Parameters and Growth Performance in Cattle: A Preliminary Study on Its Potential as a Feed Resource

**DOI:** 10.3390/ani15060788

**Published:** 2025-03-11

**Authors:** Dongwang Wu, Kai Wang, Ying Lu, Zhendong Gao, Yuqing Chong, Jieyun Hong, Jiao Wu, Weidong Deng, Xiaoming He, Dongmei Xi

**Affiliations:** 1 Yunnan Provincial Key Laboratory of Animal Nutrition and Feed Science, Faculty of Animal Science and Technology, Yunnan Agricultural University, Kunming 650201, China; danwey@163.com (D.W.);; 2 Honghe Hani and Yi Autonomous Prefecture Animal Health Supervision Institute, Honghe 661099, China; 3 Institute of Animal Husbandry, Yunnan Vocational College of Agriculture, Kunming 650201, China

**Keywords:** *Panax notoginseng*, rumen bacteria, Wenshan cattle, feed, growth performance

## Abstract

This study aimed to investigate the effects of adding different proportions of *Panax notoginseng* residue (PNR) to the diet on the rumen microbial community structure, blood biochemical indices, and growth performance of Wenshan cattle. Fifteen Wenshan cattle with an average weight of 392.30 ± 22.57 kg were randomly divided into three groups, a control group, a 3% PNR group, and a 6% PNR group, with five cattle in each group, for a 100-day feeding trial. The results show that adding PNR to the diet modulates the abundance and diversity of rumen microorganisms in Wenshan cattle, primarily affecting the relative abundances of key bacterial phyla such as Firmicutes, Proteobacteria, and Bacteroidetes. At the genus level, the relative abundances of Fibrobacter and Butyrivibrio exhibited trends of either decreasing and then increasing or increasing then decreasing with the amount of PNR added, indicating a complex regulatory effect of PNR on the rumen microbial community. The addition of PNR decreased blood glucose and blood lipid levels in Wenshan cattle. Moreover, PNR addition also increased the average daily weight gain of Wenshan cattle, demonstrating its positive effect on enhancing growth performance. In summary, PNR, as a feed resource, has potential application value in the feeding of Wenshan cattle. It not only regulates the rumen microbial community structure and improves metabolic health but also effectively enhances animal growth performance.

## Simple Summary

This study investigated the effects of different proportions of *Panax notoginseng* residue (PNR) on the rumen microorganisms, blood biochemical parameters, and growth performance of Wenshan cattle. The results showed that PNR affected rumen microbial abundance, resulting in both increases and decreases in specific microbial populations, decreased blood glucose and blood lipids, and increased average daily gain of Wenshan cattle. The PNR as a feed had positive significance in improving the production performance and physiological health of the host.

## 1. Introduction

As people’s living standards improve, the demand for meat and dairy products continues to rise, driving the rapid development of the livestock industry [1,2]. The increase in livestock products necessitates more feed for animals, which in turn intensifies competition between humans and animals for food resources, particularly grains [3]. As this competition becomes more intense, it has become urgent to find alternative non-grain plant resources to alleviate feed supply pressure, reduce basic feed costs, and promote the healthy and sustainable development of the livestock industry [4,5]. With the expansion of the livestock industry, the shortage of feed resources and rising costs have become key factors limiting the industry’s development [6,7]. Utilizing new feed resources can partially alleviate this issue by reducing the competition between humans and animals for food [8].

*Panax notoginseng* residue (PNR), a by-product of the medicinal herb *Panax notoginseng*, remains rich in crude protein, crude fat, starch, and various bioactive components even after the extraction of ginsenosides, showing great potential as a new feed resource. Researchers have conducted studies on the effects of ginseng extracts on animal performance and product quality, providing valuable insights [9,10]. Exploring and effectively utilizing PNR and other new feed resources has become an important way to alleviate feed supply pressure and promote the green development of the livestock industry. However, there are still limited studies on the application of PNR in ruminant feed and its effects on animal health and production performance. Previous studies have shown that using new feed resources can improve ruminal degradation and nutrient absorption in dairy cattle, thereby enhancing production efficiency [11].

This study hypothesizes that PNR, when used as a new feed in the feeding of Wenshan cattle, can improve their rumen microbial community structure, optimize blood biochemical parameters, and thus enhance production performance. To test this hypothesis, this study will systematically explore the potential effects of PNR on the rumen microbiota, health status, and production efficiency of ruminants. By revealing the value of PNR as a feed resource, this study aims to provide scientific evidence for optimizing feeding strategies in ruminants, improving health and production efficiency, while contributing new solutions for the sustainable development of the livestock industry and human nutritional security.

## 2. Materials and Methods

### 2.1. Experimental Design

A continuous 100-day feeding trial was conducted at Guduo Agriculture and Animal Husbandry Co., Ltd. (Wenshan, China), starting on 24 July 2021. Fifteen Wenshan cattle (bullocks), aged 30 months with a body weight of 392.30 ± 22.57 kg, were selected based on health and body condition to minimize baseline variability. The study employed a single-factor, randomized experimental design, with cattle randomly assigned to one of three groups: the D group (0% PNR replacement), the S3 group (3% PNR replacement), and the S6 group (6% PNR replacement). A computer-generated random number list ensured unbiased allocation, with five replicates per group. To evaluate the effect of PNR, it replaced corn silage and dry rice straw in the diet of the experimental cattle. Detailed dietary compositions are provided in Table 1.

To control for potential confounding factors, all cattle were kept under identical housing conditions with uniform environmental settings, including consistent ventilation and natural lighting. Feeding schedules and handling procedures were standardized across all groups to eliminate the influence of external variables. The cattle were housed in separate enclosures for each group to prevent cross-contamination of feed and to ensure that all animals within a group received identical treatment. The diet prior to the trial consisted of dry straw, whole silage corn, and crushed corn for all groups, and the pre-feeding adaptation period lasted for 10 days. This allowed the animals to acclimatize to their new diets and corral before the regular 90-day feeding phase began. Blinding was implemented during data collection and analysis to further reduce bias. The personnel responsible for collecting weight data and monitoring the cattle’s health were unaware of the group assignments. This was achieved by assigning a code to each group that only the trial manager knew, thus maintaining blinding throughout the trial period. In addition, laboratory staff conducting blood biochemical analyses were blinded to the treatment groups to avoid bias in interpreting results. Any issues were addressed promptly to ensure animal welfare and the integrity of the experiment. The only variable manipulated was the level of PNR in the concentrate, with all other feeding and management protocols kept consistent across groups. At the end of the experimental period, blood samples (15 mL) were collected from the tail vein of the cattle at 08:00. The samples were centrifuged at 2100× *g* for 15 min to separate the serum, which was then aliquoted into 1.5 mL microcentrifuge tubes and stored at −20 °C for further analysis.

Data processing was conducted using Microsoft Excel 2016. For statistical analysis, SPSS 25.0 was used. Growth performance and blood biochemical parameters were analyzed using one-way ANOVA to assess the effects of different PNR doses. The residuals were checked for normality and homoscedasticity using the Shapiro–Wilk test and Levene’s test, respectively. Multiple comparisons of mean values were performed using the LSD and Duncan methods. Additionally, linear and quadratic trends of PNR doses were evaluated using polynomial regression analysis. *p*-values less than 0.01 were considered highly significant, while *p*-values greater than 0.05 indicated no significant differences. For microbiome data, we employed PERMANOVA (Permutational Multivariate Analysis of Variance) to assess the effects of PNR doses on the rumen microbial community structure. Principal coordinate analysis (PCoA) based on Bray–Curtis distances was performed to visualize differences among groups. *p*-values less than 0.01 were considered highly significant, while *p*-values greater than 0.05 indicated no significant difference. The concentrate feed was provided at 0.8% of body weight, supplemented with mineral salts and vitamins. Diets were offered ad libitum, with feed residues collected and weighed daily to determine feed intake. Cattle were weighed on three consecutive days at the beginning and end of the trial before morning feeding to minimize measurement error. Nutritional content was evaluated using Zhang Liying’s methodology [12], assessing dry matter (DM), crude fat (EE), crude protein (CP), neutral detergent fiber (NDF), and acid detergent fiber (ADF). Starch content was analyzed using the enzymatic colorimetric method based on McCleary et al. [13]. Blood biochemical parameters were determined following the methods of the Kunming Jinyu Medical Research Institute, with all indicators measured using a Roche automatic blood analyzer (Roche, Basel, Switzerland). The biochemical kit used was from Roche (Basel, Switzerland).

The basic diet was formulated based on the 2004 edition of the Nutritional Requirements and Feeding Standards of Beef Cattle in China, adapted for local production practices. The base diet composition and its nutritional constituents are presented in Table 1. The composition content of PNR is shown in Table 2. The cattle were each fed a daily ration consisting of 1.5 kg of dry straw and 8 kg of corn silage, cut to lengths of 3–5 cm.

### 2.2. Rumen Microbial Sequencing Analysis

After the end of the experiment, the Wenshan cattle were slaughtered and their rumen contents were collected, filtered by 4 layers of gauze, divided into 15 mL sterilized centrifuge tubes, and then stored in liquid nitrogen. After extraction of genomic DNA in the sample with a MicroSEQ™ (Waltham, MA, USA)whole-gene 16S rDNA sequencing kit, amplification of conserved regions of rDNA was performed with specific primers with barcodes 341F “CCTACGGGNGGCWGCAG”, 806R, and “GGACTACHVGGGTATCTAAT” [14]. After the raw reads were obtained by sequencing, low-quality reads were first filtered and then assembled. The double-ended reads were spliced into tags, and then the tags were filtered. The resulting data were referred to as clean tags. Then, clustering was carried out based on clean tags, and chimeric tags detected in the process of clustering were removed. Finally, the data obtained were effective tags. After Operational Taxonomic Unit (OTU) values are obtained, OTU abundance statistics are carried out based on effective tags. When constructing OTUs/ASVs (Amplicon Sequence Variants), representative sequences were chosen. The RDP Classifier’s Naive Bayesian (Michigan State University, East Lansing, MI, USA) assignment algorithm was employed to label species using the database. The species with the highest average abundance across all samples was singled out for thorough presentation. The remaining species were collectively placed under the “Other” category. Tags that could be annotated to this level were categorized as “Unclassified”. The Pearson correlation coefficient among species was computed using the R language psych package (version 1.8.4), employing the species abundance table as a foundation. The *p*-value is derived through the application of the Fish-Z transform. The default choice for correlation selection is cor > 0.5. A relational pair of 0.5 is adopted as the criterion for network visualization using graphs. For the analysis of differential groups, the LEFse software (version 1.1.2) was utilized. Drawing upon the outcomes of the Kruskal–Wallis test for difference analysis, a tripartite graph (facilitated by the R language ggtern package version 3.1.0) was employed to illustrate the pronounced enrichment of each group (*p* < 0.05) in terms of the number of species, relative species abundance, and other pertinent information. To illustrate the PCoA graph, the R language ggplot2 package (version 2.2.1) was employed, while Qiime (version 1.9.1) underpins the alpha diversity analysis. The mapping of species distribution was executed using circos (version 0.69-9).

## 3. Results

### 3.1. Effects of Panax notoginseng on Blood Biochemical Parameters and Body Weight of Wenshan Cattle

The impact of PNR on the blood biochemical parameters of Wenshan cattle is shown in Table 3. In comparing the S3 group and the S6 group, within the S3 group, the cholesterol (CHOL) and low-density lipoprotein cholesterol (LDL-CH) levels were higher in the S3 group than in the S6 group (*p* < 0.05). Among the three groups, the triglyceride (TG) level in the S3 group was lower than in both the D and S6 groups (*p* < 0.05). No noteworthy differences were found in serum albumin (ALB), globulin (GLOB), direct bilirubin (DBIL), total bilirubin (TBIL), and total protein (TP) among the serum biochemical indexes of Wenshan cattle (*p* > 0.05). The glutamyl transpeptidase (GGT) level was higher in the S6 group than in the D and S3 groups (*p* < 0.05). Alanine aminotransferase (ALT) content in the S3 group was higher than that in the D and S6 groups (*p* < 0.05). Indirect bilirubin (IBIL) content in both the S3 and S6 groups was elevated in comparison to the D group (*p* < 0.05). Among the diverse impacts of different proportions of PNR on urea and blood sugar indexes of Wenshan cattle, it was evident that PNR had no discernible effect on the urea level of Wenshan cattle. The glucose (GLU) level was higher in the D group than in the S3 and S6 groups (*p* < 0.05). The S3 group showed a higher average daily gain (ADG) compared to the D group (*p* < 0.05), as shown in Table 4. Based on the data presented in Table 4, the effect of *Panax notoginseng* residue on the body weight and feed intake of Wenshan cattle is evident. The pretrial weights of the D, S3, and S6 groups were similar, indicating no significant initial differences among the groups. At the end of the trial, the post-test weights showed an increasing trend across groups, with the S3 group achieving the highest weight (448.25 ± 5.38 kg), followed by the S6 group (443.34 ± 4.56 kg) and the D group (438.84 ± 7.61 kg). The average daily feed intake (ADFI) was consistent across all groups, with values around 9.00 kg/day, indicating that feed consumption was not significantly affected by the treatment. However, differences were observed in the average daily gain (ADG). The S3 group demonstrated a significantly higher ADG (0.86 ± 0.05 kg/day) compared to the D group (0.78 ± 0.07 kg/day, *p* < 0.05), while the S6 group had an intermediate ADG (0.82 ± 0.08 kg/day) that was not significantly different from either the S3 or D groups. These results suggest that supplementation with *Panax notoginseng* residue, particularly at the S3 level, positively impacts growth performance in Wenshan cattle.

### 3.2. Rumen Microbial Sequencing Results

Through high-throughput sequencing of microbial samples from the rumen fluid of 15 Wenshan cattle, a total of 1,875,604 raw tags (representing the original tags obtained after overlap assembly) were procured. Subsequent to sequencing data screening and quality control, an average of 123,786 clean tags (representing high-quality tags after tag quality control) were generated per sample. Following the elimination of low-quality sequences, a cumulative sum of 1,629,997 effective tags (representing high-quality tags after the removal of chimeras, i.e., tags viable for subsequent analysis) were acquired. For each sample, an average of 108,666.5 effective tags were utilized in the sequencing data analysis (as indicated in Supplementary Table S1). The proportion of effective microbial sequences in the rumen of Wenshan cattle demonstrated no statistically significant differences among all groups (*p* > 0.05). To gauge microbial diversity richness, the number of OTUs was contrasted across different experimental groups. This comparison illustrated that the quantity of OTUs in groups D and S6 was notably higher than that in the S3 group (*p* < 0.05).

### 3.3. Alpha Diversity Analysis

**Figure 1:**
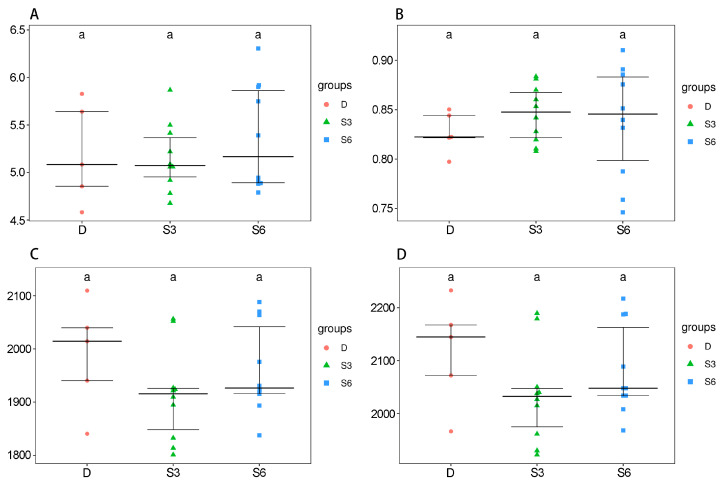
Tukey HSD test box plots are depicted in the diagram. The horizontal axis signifies the grouping, while the vertical axis represents the extent of the diversity index. The midpoint symbolizes the sample. Matching letters indicate a noteworthy disparity, whereas distinct letters signify an absence of considerable differences. The labels correspond to specific diversity indices: (**A**) Shannon, (**B**) Simpson, (**C**) Chao, and (**D**) Ace. The X-axis represents the experimental groups; the Y-axis represents a specific metric related to diversity (Shannon, Simpson, Chao, and Ace).

Alpha diversity refers to the variety and abundance of species within a specific habitat or ecosystem, providing insights into species distribution and ecological balance. It is commonly assessed using two key metrics: species richness (the number of distinct species) and species evenness (how evenly individuals are distributed among those species). The alpha diversity index for rumen microbial communities in Wenshan cattle demonstrated noteworthy discrepancies across the three groups (Figure 1). The Shannon and Simpson indices provide a holistic depiction of species richness and evenness. A larger Shannon index corresponds to a smaller Simpson index and thus signifies heightened diversity. Meanwhile, the Chao1 and Ace indices predominantly reflect the richness of species within samples, with larger values indicative of greater diversity. The coverage index calculates the proportion of low-abundance OTUs within samples, giving insight into the quantity of sequencing data. Larger values correspond to more comprehensive sequencing data coverage. Simpson index values for rumen microorganisms displayed no considerable differences among all groups (*p* > 0.05). The rumen microbial Shannon index within group D exceeded that of group S6 (*p* > 0.05), notably surpassing the S3 group (*p* < 0.05). The Chao1 and Ace indices for rumen microorganisms within groups D and S6 were higher than those within the S3 group, with these differences being statistically significant (*p* < 0.05). In summation, group S6 demonstrated the highest richness and uniformity of microbial species within the rumen fluid of Wenshan cattle.

### 3.4. Analysis of Microbial Abundance and Composition in Rumen

**Figure 2:**
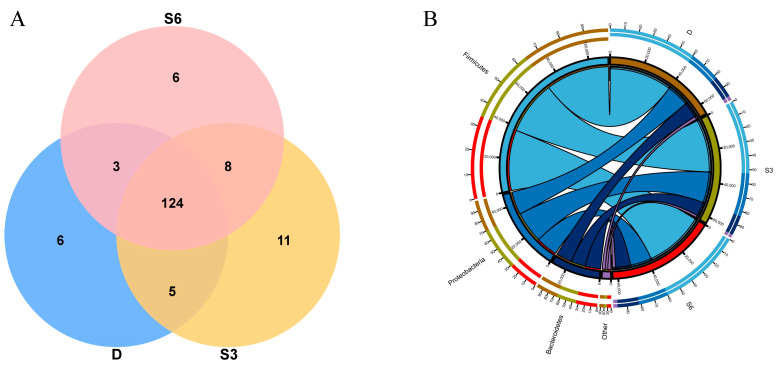
(**A**). Effect of PNR on rumen microbial OTUs of Wenshan cattle. (**B**) Circos diagram based on phylum level. The left semicircle illustrates species, while the right semicircle denotes samples/groups. The line’s thickness from species to grouping conveys the relative abundance of species within the grouping/sample. Along the outer rim of the left semicircle, colors correspond to different groups, and the length of the ring conveys the abundance of each species within each group. Similarly, along the outer rim of the right semicircle, colors signify different species, and the length of the ring conveys the abundance of each species within each group.

By utilizing Venn diagram analysis and comparing shared and exclusive species within each group, we aimed to grasp the nuances in species composition across groups. As depicted in the Venn diagram (Figure 2A), a collective tally of 163 microorganism species were detected within the rumen of Wenshan cattle across the three groups. Among these, 124 species were found to be common among all three groups, while groups D, S3, and S6 possessed 6, 11, and 6 unique species, respectively. In summation, the S3 group stands out with the highest count of both total species and exclusive species. Hence, in comparison to all other groups, the S3 group exhibits the most extensive array of species as well as the largest number of endemic species.

### 3.5. Rumen Microbial Community Structure

**Figure 3:**
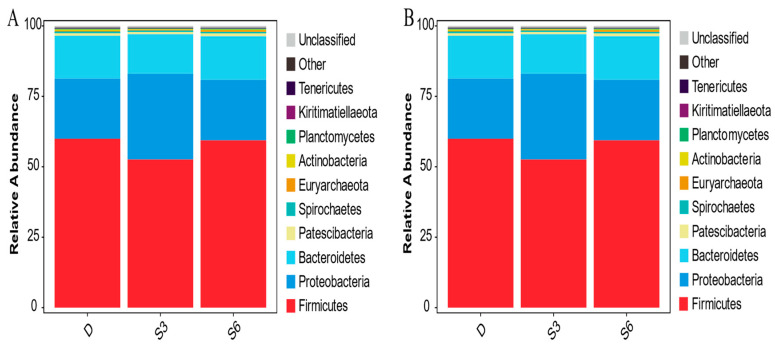
(**A**) Stacking diagram of species distribution at the phylum level of rumen bacteria in Wenshan cattle. (**B**) Distribution of species at the genus level of rumen bacteria in Wenshan cattle.

Distinct ecological environments give rise to varying microbial composition structures. Observing dissimilarities within the microbial composition of group samples holds paramount importance as it guides subsequent endeavors like mining and screening for indicator species within the samples. The Circos diagram, depicting species composition, elucidates the composition relationship through the representation of sample–species connections. By assessing the thickness of the respective lines (Figure 2B), one can gain insight into the proportion of species in the relevant sample or the proportion of a species within each sample. Dominant species play a pivotal role in shaping the ecological and functional makeup of the microbial community. Grasping the species composition within the community across different tiers facilitates a comprehensive interpretation of how the community structure takes shape, evolves, and influences the ecosystem. The tally of species composition for each sample at every classification level was tabulated. Subsequently, the visual representation of species abundance across diverse samples at each classification level took the form of a stacked graph. In Figure 3A, it becomes evident that *Firmicutes*, *Proteobacteria,* and *Bacteroridetes* are the dominant bacteria, and the abundance of the three bacteria combined is about 90%, and the rest bacteria account for a relatively low proportion, while other bacterial groups maintain a relatively lower proportion. The stack diagram, illustrating species distribution at the level of rumen bacteria in Wenshan cattle, highlights that *Solibacillus* and *Acinetobacter* are the predominant bacterial genera across all groups. Meanwhile, the proportion of other bacterial genera remains comparably lower (Figure 3B).

Figure 3 presents the relative abundance of rumen bacteria in Wenshan cattle for each group. It is evident from the table that *Firmicutes*, *Proteobacteria*, and *Bacteroidetes* are the primary bacteria in the rumen of Wenshan cattle across the three groups. The relative abundance of these dominant bacteria ranges from 12% to 64%. Meanwhile, the relative abundance of *Patescibacteria* falls between 0.7% and 1.5%. Other phyla, however, exhibit a relative abundance of less than 1%. In groups D and S6, both *Firmicutes* and *Tenericutes* show higher relative abundances compared to the S3 group (*p* < 0.05). Conversely, the relative abundance of *Proteobacteria* in the S3 group is notably higher than in groups D and S6 (*p* < 0.05).

Table 5 displays the relative abundance of rumen bacteria in Wenshan cattle within each group. It is apparent from the table that the dominant bacteria in Wenshan cattle across all groups are *Solibacillus* and *Acinetobacter*, with their relative abundances ranging from 15% to 38.5% in each respective group. The relative abundance of *Comamonas* is higher in groups D and S6 than in the S3 group (*p* < 0.05). Similarly, the relative abundance of *Acinetobacter* in group S3 is higher than that in group D (*p* < 0.05) and higher in group S3 than in group S6 (*p* < 0.01). Additionally, the relative abundance of *Tutricomonas* in the S3 group is higher than in groups D and S6 (*p* < 0.01). No significant differences in relative abundance are observed among other bacterial genera within each group (*p* > 0.05). These findings collectively illustrate that the inclusion of two proportions of PNR has discernible effects on different levels of rumen microbial flora in Wenshan cattle.

### 3.6. Differential Microbiological Analysis

**Figure 4:**
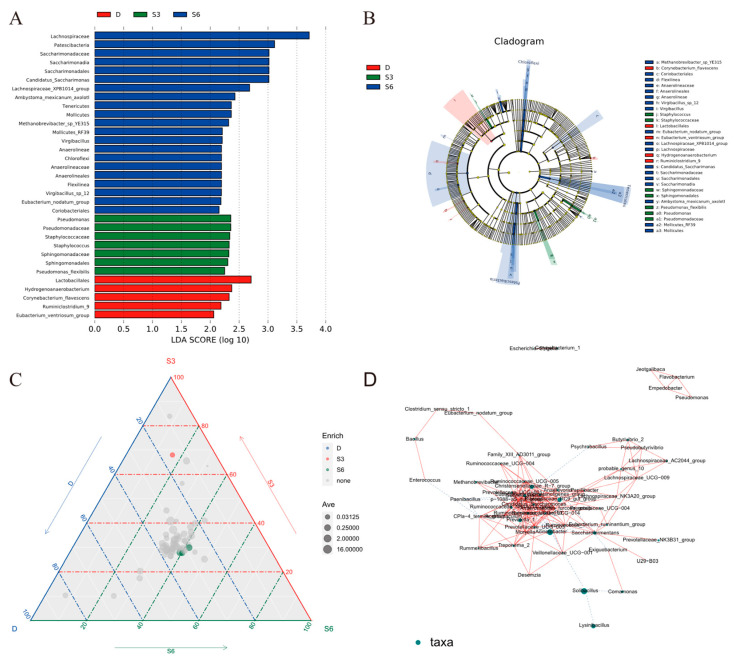
LEFse difference analysis graph. (**A**) shows species that vary in abundance in different groups, and the length of the bar chart represents the impact of different species (LDA Score). (**B**) maps the differences to a classification tree with a known hierarchy to obtain an evolutionary branching map. (**C**) Horizontal ternary diagram of genus. In the equilateral triangular coordinate system, the three endpoints refer to the three groups, the dots in the coordinate system represent the species, the color of the dots represents the significant enrichment in the corresponding color group, and the gray dots represent the species with no significant difference. The size of the dots represents the average relative abundance of the species in the three groups, and the mean is z-score homogenized to avoid the influence of extreme values. (**D**) Species correlation network graph at genus level.

LEfSe serves as a tool for the exploration and interpretation of biosignatures within high-dimensional data. It not only facilitates simultaneous differential analysis across all classification levels but also aids in identifying robust differential species between groups, commonly referred to as marker species. Figure 4A illustrates the distribution of LDA (Linear Discriminant Analysis) values for samples within each group, ranging from phylum to genus level. The outcomes reveal that certain species exerted substantial influence on the community structure within the control group. These species include *Lactobacillales*, *Hydrogenoanaerobacterium*, and *Corynebacterium_flavescens*, among others. Within the S3 group, species impacting the floral structure at the family level encompassed *Pseudomonadaceae* and *Staphylococcaceae*. Similarly, in the S6 group, species with a significant influence on the floral structure consisted of *Lachnospiraceae*, *Patescibacteria*, *Saccharimonadaceae*, *Saccharimonadales*, and *Saccharimonia*. Figure 4B presents a depiction where concentric circles illustrate the diversity of microbes from phylum to genus (or species). Notably, group S6 showcases the highest microbial diversity, followed by group S3. In the ternary plot (Figure 4C), the difference analysis, based on the Kruskal–Wallis test, revealed significant enrichment of the genus *Pseudomonas* (*p* < 0.05) within the S3 group. Similarly, within the S6 group, three genera—*Candidatus_Saccharimona*, *Lachnospiraceae_XPB1014_group*, and *Eubacterium_nodatum*—were notably enriched (*p* < 0.05). The Pearson correlation coefficient (cor > 0.5) between species was calculated using the R language psych package based on the species abundance table. The top 10 relation pairs are *Christensenellaceae_R-7_group* and *Eubacterium_coprostanoligenes_group*, *p-1088-a5_gut_group*, *Prevotella_1* and *Prevotellaceae_UCG-003*, *Succiniclasticum* and *p-1088-a5_gut_group*, *Ruminococcaceae_NK4A214_group* and *Candidatus_Saccharimonas*, *Eubacterium_coprostanoligenes_group* and *Ruminococcaceae_UCG-010*, *p-1088-a5_gut_group*, *Ruminococcaceae_UCG-010,* and *p-1088-a5_gut_group*, *Corynebacterium_1* and *Escherichia-Shigella*, and *Flavobacterium* and *Empedobacter* (Figure 4D).

## 4. Discussion

*Panax notoginseng*, one of the most important medicinal plants, leaves behind a residue (PNR) rich in components after the extraction of its active ingredients. If PNR can be developed as a new feed, like other plant residues, it could positively impact the sustainable development of Wenshan cattle farming and enhance the utilization efficiency of *Panax notoginseng* resources. This study investigates the effects of incorporating PNR into the diet of Wenshan cattle to explore its potential as a new feed.

The nutritional composition of the diet affects the growth performance of beef cattle, primarily reflected in the average daily gain (ADG) [15]. Therefore, improving feed conversion efficiency and growth performance is key to increasing beef cattle farming benefits [16]. PNR contains abundant nutrients, particularly high starch content, which can meet the nutritional needs for cattle growth. Also, PNR contains crude polysaccharides, saponins, and other bioactive components that can regulate the gastrointestinal microbiota, enhance digestion and absorption, improve metabolism, and increase feed digestibility, thereby improving the growth performance of Wenshan cattle [17]. The results show a quadratic relationship between PNR supplementation and growth performance. The S3 group (3% PNR) exhibited the highest final weight and ADG, while the S6 group (6% PNR) showed a slight decrease, suggesting potential diminishing returns at higher doses. Importantly, feed intake remained unchanged across groups, indicating that PNR likely enhances nutrient utilization rather than increasing feed consumption. These findings suggest that 3% PNR supplementation is optimal for improving growth performance in Wenshan cattle.

Blood lipid and serum biochemical parameters are important indicators of animal health, reflecting feed utilization, nutrient absorption, metabolism, and changes in organ function [18,19]. Alkaline phosphatase (ALP), highly expressed in mineralized tissue cells, plays a crucial role in hard tissue formation [20]. *Panax notoginseng* saponins have notable effects on cardiovascular, central nervous, and immune systems, including anti-myocardial ischemia, anti-inflammatory, and immune-enhancing effects [21]. These effects may be linked to the regulation of serum biochemical indices and metabolic functions in Wenshan cattle, potentially through the modulation of genes related to serum biochemical metabolism and enzyme activity [22,23]. We analyzed the linear and quadratic effects of PNR supplementation and observed significant dose-dependent trends in key biochemical parameters. The quadratic effects on cholesterol, LDL-C, TG, ALT, and ALP suggest that moderate PNR supplementation (3%) optimally regulates lipid metabolism and liver function, whereas excessive inclusion (6%) may trigger metabolic adaptations. The linear decrease in AST and glucose levels indicates a beneficial effect of PNR on energy metabolism, while the linear increase in GGT and IBIL suggests potential metabolic stress at higher doses. These findings highlight the need for optimized dosing strategies when incorporating PNR into cattle diets to maximize benefits while minimizing potential metabolic burdens.

The impact of PNR supplementation on the rumen microbiota followed a quadratic pattern, with S3 (3% PNR) promoting beneficial bacterial genera while S6 (6% PNR) led to a partial reversal of these effects. Acinetobacter and Comamonas were significantly enriched in the S3 group, suggesting that moderate PNR supplementation optimally supports bacterial communities involved in fiber degradation and metabolic activity. Solibacillus abundance decreased in S3 but rebounded in S6, indicating a potential adaptive shift in microbiota composition at higher PNR levels. The observed microbial changes align with improvements in nutrient absorption and metabolic efficiency, supporting the observed increase in average daily gain (ADG) in the S3 group. Notably, PNR supplementation significantly impacted blood glucose levels, with the D group showing higher levels compared to the S3 and S6 groups. This suggests that PNR may help regulate blood glucose, which could have implications for managing metabolic health in ruminants. The addition of new herbal feeds to diets can influence gut microbiota changes [24,25,26]. *Panax notoginseng* contains various effective components, including saponins, notoginsenosides, flavonoids, polysaccharides, fatty acids, and cyclic peptides. The rumen microbiome is a rich source of enzymes for degrading complex plant polysaccharides. At the phylum level, the dominant bacterial phyla in all groups were Firmicutes, Proteobacteria, and Bacteroidetes. The addition of 3% PNR decreased the abundance of Firmicutes (*p* < 0.05) and increased the abundance of Proteobacteria (*p* < 0.05). Evans et al. [27] found that Firmicutes primarily decompose cellulose, and dysbiosis of the colonic microbiota is often associated with increased Proteobacteria abundance. Proteobacteria is one of the most diverse bacterial phyla [28]. Dietary changes can alter rumen microbiota, and host adaptations to dietary diversity can affect feed efficiency. Research indicates that Solibacillus is related to dietary intake types and may increase in abundance with diverse food resources [29,30]. The abundance of Solibacillus in the S3 group was lower than in the D and S6 groups, potentially indicating an adaptive response to PNR. Acinetobacter has been found to positively correlate with milk protein content in the rumen of certain animals [31]. In pandas, dietary modifications have been shown to increase the abundance of bacterial genera such as Acinetobacter, Clostridium, and Pseudomonas [32]. These bacteria are likely involved in breaking down fibrous components of the diet, including cellulose, hemicellulose, and lignin. Similarly, in ruminants, the rumen microbiota play an essential role in facilitating the digestion of fiber and contributing to overall host metabolism. In this study, the S3 group showed significant enrichment of Acinetobacter and Pseudomonas, suggesting that PNR addition might affect the rumen microbiota’s ability to degrade lignin and improve production performance. Overall, this study provides a potential strategy for enhancing animal production performance through the use of PNR.

## 5. Conclusions

In summary, PNR demonstrates significant potential as a novel feed resource for Wenshan cattle. In this study, a 3% PNR supplementation level was found to be the most suitable, as it effectively modulated the rumen microbial community structure, increased microbial diversity, improved host metabolic health, and enhanced growth performance. Future research should further investigate the impact of PNR on other ruminants to validate its applicability and potential value on a broader scale.

## Figures and Tables

**Table 1:** Composition of basic diet.

Items	D	S3	S6
Corn	15.60	15.60	15.60
Soybean meal	5.00	5.00	5.00
Barley	3.30	3.30	3.30
Wheat bran	2.10	2.10	2.10
Premix (1)	3.00	3.00	3.00
Molasses	3.00	3.00	3.00
NaCl	1.00	1.00	1.00
NaHCO_3_	1.00	1.00	1.00
Corn silage	50.00	48.00	46.00
Dry rice straw	16.00	15.00	14.00
PNR	0.00	3.00	6.00
Total	100.00	100.00	100.00
Nutrient levels (2)			
NEmf/(MJ/kg)	10.92	10.88	10.85
CP	13.41	13.30	13.26
EE	4.31	4.28	4.25
Starch	26.19	25.67	25.17
NDF	34.26	33.30	34.52
ADF	17.51	17.53	17.60
Ca	0.92	0.87	0.90
P	0.56	0.54	0.50

Note: (1) The content in premix contains various necessary vitamins and trace elements. The chemical composition of the two kinds of diet mainly contains vitamin A 200 ku/kg, 340 ku/kg of vitamin D3, vitamin E 600 mg/kg, nicotinic acid 400 mg/kg, manganese 700 mg/kg, Se 2 000 mg/kg, zinc 460 mg/kg, iron 340 mg/kg, copper 500 mg/kg, iodine 6 mg/kg, selenium 25 mg/kg, and cobalt 5 mg/kg. (2) NEmf was a calculated value, while the others were measured values.

**Table 2:** Composition content of *Panax notoginseng* residue (%).

Items	Content (%)
Ash	8.35
CP	8.75
EE	0.53
Starch	16.00
CF	15.3
ADF	25.3
NDF	45.1
Crude polysaccharide	1.20
Total flavones	<0.01
Total saponins	0.01

**Table 3:** Influence of different proportions of PNR on blood biochemical parameters of Wenshan cattle.

Index	D	S3	S6	*p*-Value
CHOL (mmol/L)	2.79 ± 0.89 ^ab^	3.20 ± 0.39 ^a^	2.59 ± 0.32 ^b^	0.047
LDL-CH (mmol/L)	0.80 ± 0.35 ^ab^	1.08 ± 0.12 ^a^	0.61 ± 0.25 ^b^	0.043
TG (mmol/L)	0.24 ± 0.10 ^a^	0.12 ± 0.04 ^b^	0.21 ± 0.10 ^a^	0.041
HDL-CH (mmol/L)	1.67 ± 0.37	1.76 ± 0.24	1.73 ± 0.24	0.807
GGT (U/L)	16.64 ± 6.86 ^a^	16.61 ± 6.36 ^a^	21.52 ± 2.83 ^b^	0.037
ALB (g/L)	38.91 ± 3.55	38.90 ± 1.60	39.13 ± 1.81	0.987
ALT (U/L)	27.82 ± 2.81 ^b^	31.16 ± 4.59 ^a^	24.92 ± 3.06 ^c^	0.050
IBIL (umol/L)	1.93 ± 0.68 ^a^	2.32 ± 0.76 ^b^	2.42 ± 0.60 ^b^	0.033
ALP (U/L)	2.60 ± 1.86 ^a^	1.34 ± 0.93 ^c^	1.90 ± 1.20 ^b^	0.028
GLOB (g/L)	41.03 ± 3.01	37.55 ± 4.86	39.41 ± 2.74	0.354
AST (U/L)	104.52 ± 13.88 ^a^	103.84 ± 27.90 ^a^	90.80 ± 16.87 ^b^	0.049
DBIL (umol/L)	1.90 ± 0.73	1.78 ± 0.57	2.19 ± 0.85	0.671
TBIL (umol/L)	3.83 ± 0.98	4.10 ± 1.31	4.60 ± 1.38	0.613
TP (g/L)	79.94 ± 3.21	76.45 ± 5.51	78.53 ± 1.86	0.381
UREA (mmol/L)	4.56 ± 0.80	4.36 ± 0.58	4.49 ± 0.46	0.897
GLU (mmol/L)	8.06 ± 1.74 ^a^	6.17 ± 1.31 ^b^	6.53 ± 2.09 ^b^	0.031

Note: The mean ± standard deviation was taken for each group. In the same row of data, superscript letters or the absence of the same letter indicates no significant difference (*p* > 0.05); different small letters mean significant difference (*p* < 0.05). D group (concentrate added 0% of PNR), S3 group (concentrate added 3% of PNR) and S6 group (concentrate added 6% of PNR). CHOL (cholesterol), LDL-CH (low-density lipoprotein cholesterol), TG (triglyceride), HDL-CH (high-density lipoprotein), GGT (glutamyl transpeptidase), ALB (serum albumin), ALT (alanine aminotransferase), IBIL (indirect bilirubin), ALP (alkaline phosphatase), GLOB (globulin), AST (aspartate transaminase), DBIL (direct bilirubin), TBIL (total bilirubin), TP (total protein), GLU (glucose). An enzyme unit (often abbreviated as U) is a measure of the amount of enzyme activity.

**Table 4:** Effect of *Panax notoginseng* residue on body weight of Wenshan cattle.

Items	D	S3	S6	*p*-Value
Pretrial weight (kg)	368.64 ± 6.25	370.85 ± 7.68	369.54 ± 5.76	0.832
Post-test weight (kg)	438.84 ± 7.61 ^b^	448.25 ± 5.38 ^a^	443.34 ± 4.56 ^ab^	0.026
Average daily feed intake (kg/d)	9.10 ± 0.62	9.00 ± 0.79	9.08 ± 0.46	0.675
ADG (kg/d)	0.78 ± 0.07 ^b^	0.86 ± 0.05 ^a^	0.82 ± 0.08 ^ab^	0.031

Note: D group (added 0% of PNR), S3 group (added 3% of PNR) and S6 group (added 6% of PNR). In the same row of data, superscript letters or the absence of the same letter indicates no significant difference (*p* > 0.05); different small letters mean significant difference (*p* < 0.05).

**Table 5:** Relative abundance (%) of rumen microorganisms at genus level of Wenshan cattle in each group.

Item	D	S3	S6	*p*-Value
*Solibacillus*	36.60 ± 5.73 ^a^	25.39 ± 14.98 ^b^	38.13 ± 7.79 ^a^	0.049
*Acinetobacter*	20.69 ± 14.86 ^b^	33.68 ± 11.43 ^a^	15.97 ± 4.88 ^c^	0.026
*Rikenellaceae_RC9_gut_group*	5.63 ± 1.36	5.31 ± 1.30	6.32 ± 0.92	0.656
*Lysinibacillus*	5.17 ± 2.50	7.22 ± 6.24	5.10 ± 2.17	0.081
*Christensenellaceae_R-7_group*	4.35 ± 0.81	3.82 ± 0.40	4.94 ± 0.83	0.161
*Prevotella_1*	2.91 ± 1.19	1.92 ± 0.52	2.43 ± 0.23	0.199
*Saccharofermentans*	1.59 ± 0.46	1.47 ± 0.30	1.89 ± 0.28	0.268
*Succiniclasticum*	1.56 ± 0.84	1.03 ± 0.23	1.54 ± 0.39	0.228
*Ruminococcaceae_NK4A214_group*	1.12 ± 0.24	0.94 ± 0.09	1.17 ± 0.27	0.298
*Comamonas*	0.21 ± 0.31 ^c^	1.54 ± 2.08 ^a^	0.53 ± 0.97 ^b^	0.045
Other	10.57 ± 2.31	9.30 ± 2.14	11.88 ± 1.56	0.360
Unclassified	9.58 ± 2.76	8.38 ± 0.71	10.11 ± 1.56	0.069

Note: D group (concentrate added 0% of PNR), S3 group (concentrate added 3% of PNR) and S6 group (concentrate added 6% of PNR). In the same row, data with superscript letters or the absence of the same letter indicates no significant difference (*p* > 0.05); different small letters mean significant difference (*p* < 0.05).

## Data Availability

Sequence Read Archive records will be accessible with the following link after the indicated release date: http://www.ncbi.nlm.nih.gov/bioproject/1011225 (accessed on 31 August 2023).

## Supplementary Materials

The following supporting information can be downloaded at https://www.mdpi.com/article/10.3390/ani15060788/s1: Table S1: Effect of *panax notoginseng* residue on the quality of rumen microbial sequencing in Wenshan cattle.
