# Supplementary material for: Impact of Panax notoginseng Residue on Rumen Microbial Community, Blood Biochemical Parameters and Growth Performance in Cattle: A Preliminary Study on Its Potential as a Feed Resource
Source: Animals (Basel). 2025 Mar 11;15(6):788. doi: 10.3390/ani15060788 (PMC11939262; doi:10.3390/ani15060788)
Supplement: Supplementary file 1 [file animals-15-00788-s001.zip › animals-3422225-supplementary.pdf]

Supplementary Table S1 Effect of panax notoginseng residue on the quality of rumen microbial sequencing in Wenshan cattle

Items	Group		
	D	S3	S6
Raw Reads	131304.00±4775.24	126798.80±4795.43	125539.00±6272.19
Raw Tags	128447.00±4881.30	123913.20±4872.51	122760.60±5945.93
Clean Tags	127247.00±5104.72	122629.00±4596.01	121482.00±5703.69
Chimera Tags	15614.60±1257.13	15310.80±629.36	14433.20±1642.90
Effective Tags	111632.40±4115.73	10318.20±4022.15	107048.80±4160.56
Effective Ratio (%)	85.02±0.62	84.64±0.23	85.31±1.18
OTU Numbers	1844.00±123.41a	1712.00±54.49b	1827.20±100.39a

Note: D group (concentrate added 0% of PNR), S3 group (concentrate added 3% of PNR) and S6 group (concentrate added 6% of PNR).
